# Graph neural networks with configuration cross-attention for tensor compilers

**DOI:** 10.3389/frai.2025.1605539

**Published:** 2025-08-20

**Authors:** Dmitrii Khizbullin, Eduardo Rocha de Andrade, Thanh Hau Nguyen, Matheus Pedroza Ferreira, David R. Pugh

**Affiliations:** ^1^King Abdullah University of Science and Technology (KAUST), Thuwal, Saudi Arabia; ^2^Sprout.ai, London, United Kingdom

**Keywords:** graph neural network (GNN), tensor compilation, attention mechanism, ranking loss function, machine learning for systems

## Abstract

With the recent popularity of neural networks comes the need for efficient serving of inference workloads. A neural network inference workload can be represented as a computational graph with nodes as operators transforming multidimensional tensors. The tensors can be transposed and/or tiled in a combinatorially large number of ways, some configurations leading to accelerated inference. We propose TGraph, a neural graph architecture that allows screening for fast configurations of the target computational graph, thus representing an artificial intelligence (AI) tensor compiler in contrast to traditional heuristic-based compilers. The proposed solution improves mean Kendall's τ across layout collections of TpuGraphs from 29.8% of the reliable baseline to 67.4% of TGraph. We estimate the potential CO_2_ emission reduction associated with our work to be equivalent to over 50% of the total household emissions in the areas hosting AI-oriented data centers.

## 1 Introduction

Machine learning (ML) continues to gain popularity in solving engineering tasks, including Large Language Models for natural language processing, convolutional and transformer models for computer vision, recommendation models in online services, etc. (Minaee et al., 2024; Pugliese et al., 2021). Most of the computing associated with ML is done by serving ML models for inference rather than training them (Desislavov et al., 2023). The need to reduce monetary costs as well as the CO_2_ footprint of inference workloads leads to significant efforts in the optimization of computations (Bolón-Canedo et al., 2024; Tschand et al., 2025). Typically, ML workloads are launched on specialized accelerators: GPUs, TPUs (Jouppi et al., 2023), and others, which do not provide the same level of on-chip real-time optimization as CPUs do. Consequently, the complexity of optimization of computations for ML accelerators is shifted toward the compiler. Implementation of an enormous quantity of specialized kernels supporting the full matrix formed by a variety of accelerators times a variety of ML models seems intangible (Huyen, 2024). One solution to this problem is to employ ML-based tensor compilers.

### 1.1 Related work

Several attempts have been made to build a highly efficient tensor compiler in recent years. Tensorflow (Abadi et al., 2016) has a rule-based XLA tensor program optimization engine Sabne (2020) that was studied by Snider and Liang (2023). TVM (Chen et al., 2018a) introduces a Python-based meta-language to describe the computation and its execution schedule separately, allowing a range of automated optimizations mostly limited to one operator and avoiding operator (kernel) fusion. AutoTVM (Chen et al., 2018b) introduces the optimization of tensor programs based on gradient-boosted trees and TreeGRU and uses the ranking loss for model training rather than element-wise losses like MSE. PyTorch (Paszke et al., 2019), being a framework built with the imperative paradigm in mind, in its recent version, supports TorchScript, a just-in-time (JIT) compiled for the annotated functions and classes. JAX (Bradbury et al., 2018) as a functional meta-language natively supports JIT.

TASO (Jia et al., 2019) performs equivalent graph substitution as a way to fuse kernels. PET (Wang et al., 2021) then builds on top of TASO (Jia et al., 2019) to expand the search space to non-equivalent transformations and apply automatically generated correction kernels. DeepCuts (Jung et al., 2021), Ansor (Zheng et al., 2020), and TensorComp (Vasilache et al., 2018) rely on heuristics to solve the problem of efficient execution of a computational graph. NN-Meter (Zhang et al., 2021) presents a latency prediction model based on a combination of heuristics to account for the effects of kernel fusion and a random forest for single-operator latency prediction.

A significant fraction of the aforementioned works rely solely on heuristics and rules to compile a tensor program. Although the compilation time of a heuristic-based algorithm may be very small, it fails to achieve the absolute minimum of program runtime. In this work, we propose an algorithm based on machine learning to optimize a tensor program that is represented as a computational graph. The closest works to ours are Phothilimthana et al. (2020) and Xu et al. (2023) that use the same dataset and a benchmark TpuGraphs (Phothilimthana et al., 2023). Graph Segment Training (GST) (Cao et al., 2023) uses TpuGraphs as well but reports another metric, OPA, and does not provide a breakdown across the collections.

Apart from TpuGraphs, few datasets represent runtime measurements of computational graphs: Tenset (Zheng et al., 2021) and the dataset published by the authors of nn-meter Zhang et al. (2021), while none of these explicitly organizes the node and edge attributes in a systematic way suitable for machine learning.

Traditional tensor compilers employ heuristic-based approaches to optimize computational graphs for hardware accelerators. The typical workflow involves several stages: graph analysis to identify optimization opportunities, operator fusion to combine adjacent operations into single kernels, memory layout optimization to improve data locality, and code generation targeting specific hardware. Heuristic compilers like XLA rely on predefined rules and patterns to make optimization decisions, such as choosing tensor layouts or tiling strategies based on operator types and tensor shapes. While these approaches offer fast compilation times and predictable behavior, they are inherently limited by the quality of hand-crafted rules and struggle to adapt to the diverse and rapidly evolving landscape of neural network architectures and hardware accelerators. Recent AI-based approaches aim to overcome these limitations by learning optimization strategies from data, using techniques such as reinforcement learning to explore the optimization space or supervised learning to predict the performance of different configurations. However, existing ML-based methods such as AutoTVM and Ansor focus primarily on single-operator optimization and fail to capture the complex inter-operator dependencies that arise in full computational graphs. Most critically, these approaches treat configuration optimization as an individual prediction problem, where each configuration is evaluated in isolation without explicit comparison to alternatives, ignoring the inherently relative nature of the optimization task. Our TGraph architecture addresses these limitations through two key innovations: (1) cross-configuration attention that enables explicit comparison between different configurations within the same batch, transforming the problem from individual prediction to learned ranking, and (2) a graph neural network architecture specifically designed for computational graphs with configurable nodes, allowing the model to capture both local operator behavior and global graph-level optimization opportunities while operating on pruned subgraphs for improved computational efficiency.

### 1.2 TpuGraphs dataset and benchmark details

The only publicly available dataset for the large-scale compiler configuration search is TpuGraphs (Phothilimthana et al., 2023). TpuGraphs contains execution times of an XLA's HLO graph with a specific compiler configuration on a Tensor Processing Unit (TPU v3). TpuGraphs focuses on optimizing tensor layouts and tensor tiling as compiler configurations. A tensor layout describes the order in which dimensions of a tensor are arranged or permuted in memory. Specifically, a layout is a 0-based sequence of dimension indices. A tensor tiling configuration defines how to partition a tensor into smaller sub-tensors or tiles by specifying the ranges of indices for each dimension, enabling efficient computation and memory locality. The tensor layout optimization dataset comprises 4 collections organized in a matrix shown in Table 1. The two groups of network architectures (xla and nlp) represent two distinct categories of workloads: xla - predominantly computer vision loads, while nlp—exclusively transformer-based natural language processing loads. Each architecture has up to 100,000 different tensor layout configurations and the associated runtimes recorded. The total number of unique architectures in layout:xla collections is 78 with the average number of configurations of over 11,000 (for layout:xla::random), and in layout:nlp collections—244 with the average number of configurations of over 66,000 (for layout:nlp::random). Another dimension across which the layout dataset is organized is the utilized configuration search strategy: random or genetic-algorithm-based (GA-based, denoted as Default). Even though the final goal is to be able to predict configurations' runtimes, during the dataset creation, some sort of bootstrapping search must be used. Random search gives very wide coverage across all the possible runtimes, whereas the GA-based search focuses more on sampling runtimes in the vicinity of the fastest runtime, making the task of runtime prediction harder and very challenging for the predictive model.

**Table 1:** The matrix of the 4 Layout collections.

**Group of graphs**	**Configuration sampling strategy**	
	**Random (uniform)**	**Default (GA-based)**
XLA (CV, NLP and other)	layout-xla-random	layout-xla-default
NLP (Transformers)	layout-nlp-random	layout-nlp-default

**Figure 1:**
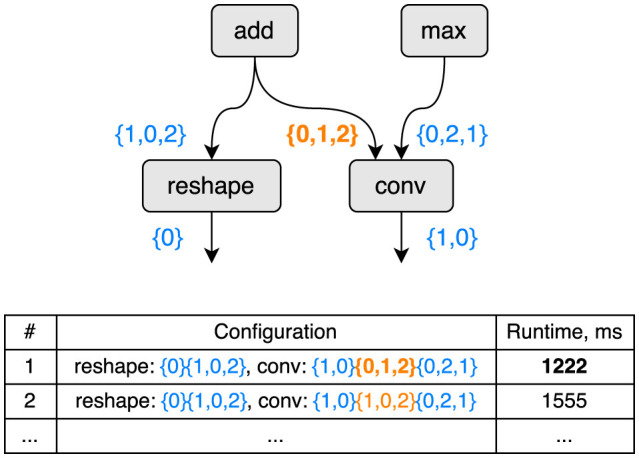
An example of how different tensor layout configurations affect the runtime of the computational (sub-)graph. Configuration 1 is faster than and, consequently, superior to configuration 2.

To illustrate the problem of configuration selection we provide an example on Figure 1. Here, four elementary operations compose a computational graph, while only two of them, reshape and conv, are configurable. A tensor layout can be chosen by the compiler, and the choice results in potentially significantly different runtimes as a result of random or sequential memory access and deep specifics of a particular computational unit. More details can be found in Phothilimthana et al. (2023) Figure 3.

### 1.3 Contribution summary

Our contributions can be summarized as follows:

- We propose TGraph, a graph neural network (GNN) architecture with cross-channel and cross-configuration attention that achieves state-of-the-art on the TpuGraphs benchmark.
- We show very efficient training and inference by applying non-configurable node pruning, configuration de-duplication, and compression.

### 1.4 Societal impact

We perform a case study to highlight the importance of data center AI workload optimization. According to our estimates the potential impact of this work can be reduction of CO_2_ emissions equivalent to 50% (or higher) of household emissions in areas similar to North Virginia, VA. The details can be found in Section 2.6.

## 2 TGraph runtime ranking architecture

### 2.1 Problem specification

We are looking to find the configuration $\tilde{c}$ that minimizes the tensor program runtime *R*(*c*) across the configuration space *C* for a specific computational graph.


(1) $$\begin{array}{l} \tilde{c}=\underset{c\in C}{\text{argmin}}\left(R\left(c\right)\right) \end{array}$$

As we have only partial knowledge of R(c) in the form of benchmarked data, we are looking for a solution as an approximation *R*_*neural*_(*c*) of the underlying true *R*(*c*).

The configuration space *C* can be described as ℤ^*N*^ where *N* is the number of discrete configurable variables (node and edge attributes) in a specific graph.

### 2.2 Data pre-processing

#### 2.2.1 Graph pruning

**Figure 2:**
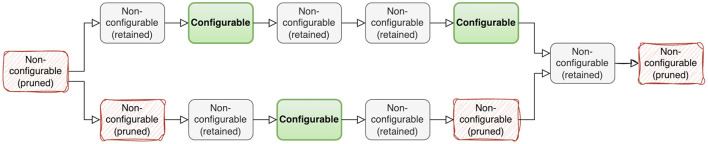
An example of node pruning. Nodes that are not connected to configurable nodes are removed (red nodes on the diagram). Two disconnected subgraphs are left after pruning.

For layout collections, only Convolution, Dot, and Reshape nodes are configurable. Also, in most cases, the majority of nodes are identical across the configuration set. Thus, we adopt the following pruning strategy: for each graph, we only keep the nodes that are either configurable nodes themselves or are connected to a configurable node, i.e., input or output to a configurable node. By doing this, we transform a single graph into multiple (possibly disconnected) sub-graphs. The possibly disconnected graph does not pose a problem since TGraph has a global graph pooling layer as one of the final layers that fuses the sub-graph information. This way of graph pruning reduces the vRAM usage 4 times and speeds up training by a factor of 5 in some cases. An example of graph pruning is shown on Figure 2.

#### 2.2.2 Configuration deduplication

Most of the configuration sets for layout collections contain a lot of duplication. The runtime for the duplicated configuration sets can vary up to 0.4% of the mean value. Training on the same configuration sets but different runtime targets makes loss noisy and the training process less stable. Thus, we remove all the duplicated configuration sets for layout collections and leave the smallest runtime value for determinism.

#### 2.2.3 Lossless configuration compression

Even with pruning and de-duplication, the RAM usage to load all configurations to the system memory for NLP collections is beyond the RAM capacity. We circumvent that issue by compressing node_config_feat beforehand and only decompressing it on the fly in the data loader after configuration sampling. This allows us to load all data to memory at the beginning of training, which reduces IO/CPU bottlenecks considerably and allows us to train faster. The compression is implemented based on the fact that each node_config_feat 6-dim vector (input, output, and kernel) can only have 7 possible values (-1, 0, 1, 2, 3, 4, 5) and, thus, can be represented by a single integer in base-7 (from 0 to 7^6^−1).

#### 2.2.4 Changing the pad value in node_feat

The features in node_feat are 0-padded. Whilst this is not a problem for most features, for others like layout_minor_to_major_*, this can be ambiguous since 0 is a valid axis index. Also, the node_config_feat are −1 padded, which makes it incompatible with layout_minor_to_major_* from node_feat. With that in mind, we re-generate node_feat with −1 padded, and this allows us to use a single embedding matrix for both node_feat[134:] and node_config_feat.

#### 2.2.5 Data normalization, embedding and batching

**Figure 3:**
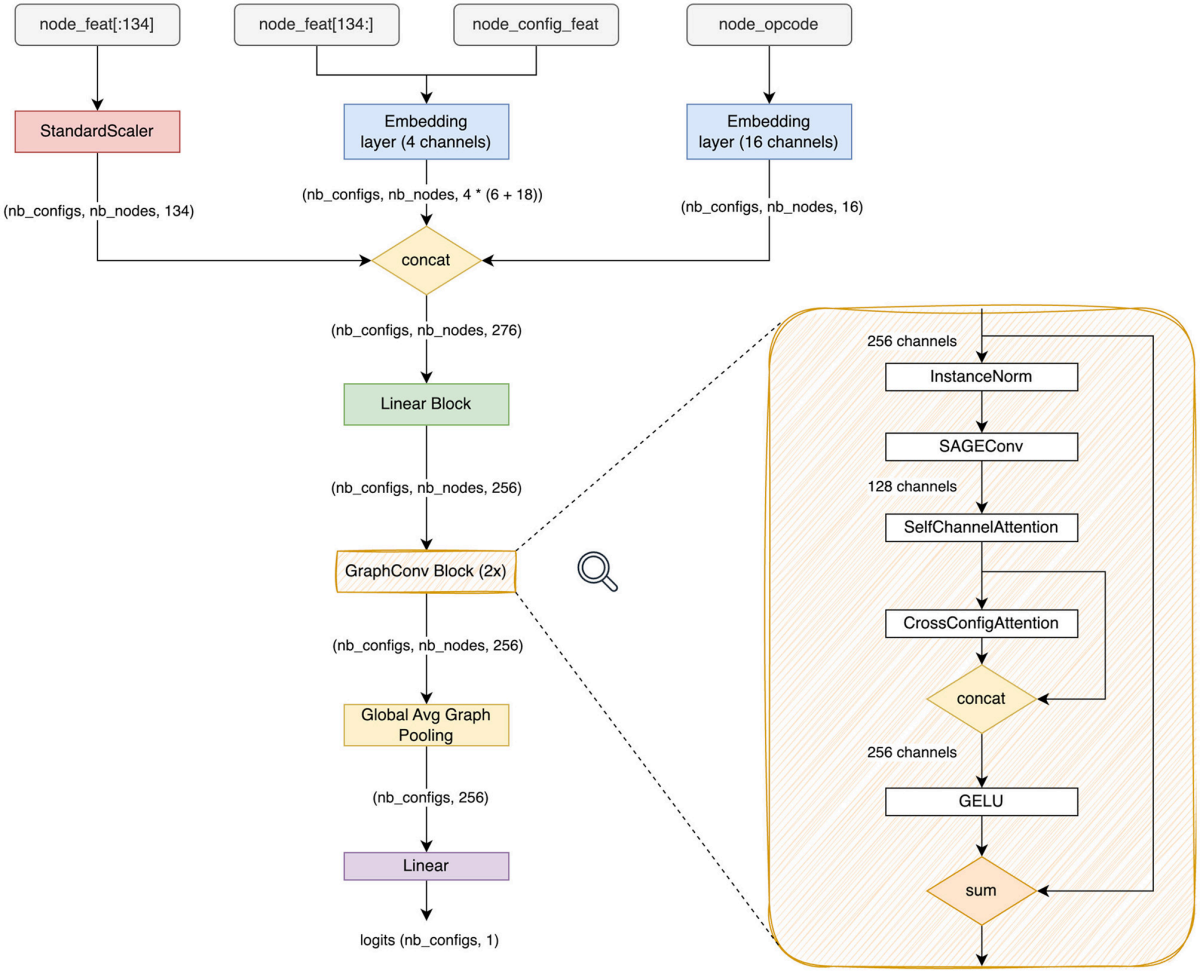
Architecture diagram of TGraph. *n*_*configs*_ is the number of configurations sampled into a batch. *n*_*nodes*_ is the number of nodes in the sampled graph after pruning.

For layout, the node features are formed as a 140-dimensional vector node_feat that represents various fields in an XLA's HLO instruction (a node in an HLO graph) either as they are, or as categorical values using one-hot encoding. We split node_feat into node_feat[:134] containing numerical and one-hot-encoded values and node_feat[134:] that contains the tensor index permutation of the output tensor layout (layout_minor_to_major_*). The former is normalized to element-wise 0-mean and unit standard deviation (StandardScaler on Figure 3), while the latter, along with node_config_feat, is fed into a learned embedding matrix (4 channels). We find that the normalization is essential since node_feat has features like *_sum and *_product that can be very high in values compared to the rest of the features and, consequently, disrupt the optimization. Further, we find that the natural way to encode the permutation vectors is to embed them into a low-dimensional vector. For node_opcode, we also use a separate embedding layer with 16 channels. The input to the network is the concatenation of all aforementioned features. For each graph, we sample on the fly a batch of 64 (for default collections) or 128 (for random collections) configurations to form the input batch. For tile, on the other hand, we opt to use late fusion to integrate config_feat into the network.

### 2.3 Architecture details

Following the reasoning laid out by (Phothilimthana et al. 2020), we employ GraphSAGE (Hamilton et al., 2017) as a basis of a graph convolutional block. GraphSage operation can be expressed as


(2) $$\begin{array}{l} S_{i}^{k}\left(\varepsilon \right)=N_{L2}\left(f_{2}^{k}\left(\text{concat}\left(\varepsilon_{i},\underset{j\in \text{neighbors}\left(i\right)}{\sum }f_{1}^{k}\left(\varepsilon_{j}\right)\right)\right)\right) \end{array}$$

where *i* is the index of a node, *k* is the index of the layer, $f_{1...2}^{k}$ - feedforward layers at the specific depth *k*, *N*_*L*2_ - *L*_2_ normalization, *neighbors*(*i*) - a set of immediate neighbors of node *i*.

We construct the graph convolutional block that can be expressed in the following way.


(3) $$\begin{array}{l} B_{i}^{k}\left(\varepsilon \right)=\varepsilon +a\left(\text{concat}\left(\eta_{i},A_{cross}\left(\eta_{i}\right)\right)\right) \end{array}$$

where *a* is GELU activation, *A*_*cross*_ - configuration cross-attention operation, and η_*i*_(ε) is expressed as:


(4) $$\begin{array}{l} \eta_{i}\left(\varepsilon \right)=A_{self}\left(S_{i}^{k}\left(N_{instance}\left(\varepsilon \right)\right)\right) \end{array}$$

Here *A*_*self*_ is the self-attention operation described below, *N*_*instance*_ is instance normalization.

#### 2.3.1 Channel-wise self-attention

Inspired by the idea of Squeeze-and-Excitation (Hu et al., 2018), we add a channel-wise self-attention layer as a part of the graph convolutional block. We first apply a Linear layer to bottleneck the channel dimensions (8x reduction), followed by ReLU. Then, we apply a second linear layer to increase the channels again to the original value, followed by sigmoid. We finish by applying element-wise multiplication to the obtained feature map and the original input. The idea behind channel-wise self-attention is to capture the correlations between channels and use them to suppress less useful ones while enhancing the important ones.


(5) $$\begin{array}{l} A_{self}\left(\varepsilon \right)=\varepsilon ○\sigma \left(f_{squeeze}\left(\text{ReLU}\left(f_{excitation}\left(\varepsilon \right)\right)\right)\right) \end{array}$$

Here ○ denotes element-wise multiplication.

#### 2.3.2 Cross-configuration attention

Another dimension in which we apply the attention mechanism is the batch dimension: across the sampled configurations. We design the cross-configuration attention block that allows the model to explicitly compare each configuration against the others throughout the network. We find this method to be much superior to letting the model infer for each configuration individually and only compare them implicitly via the loss function (PairwiseHingeLoss in this paper). The cross-configuration attention expression comes as follows:


(6) $$\begin{array}{l} A_{cross}\left(\varepsilon \right)=\varepsilon_{i}^{b}○\underset{b}{\text{Softmax}}\left(\varepsilon_{i}^{b}/T\right) \end{array}$$

Here *i* is the node index, *b* is the configuration index across the batch dimension, *T* is a learnable temperature parameter.

By applying the cross-configuration attention layer after the channel-wise self-attention at every block of the network, we observe a significant improvement of the target metric (Kendall's τ), especially for default collections.

#### 2.3.3 Entire architecture

The full architecture of TGraph is shown in Figure 3. After feature concatenation, we apply a fully-connected layer, then we apply a stack of 2 graph convolutional blocks $B_{i}^{k}$, *k*∈1..2, then we perform global average pooling over the node dimension indexed by *i*, and finally, we apply another linear layer to eliminate the feature dimension and get the vector of scores *s*_*c*_ where *c* is the index across the configuration dimension.

The entire network prediction can be expressed as:


(7) $$\begin{array}{l} R_{neural}\left(X\right)=f_{out}\left(Pool_{global}\left(B_{2}\left(B_{1}\left(f_{in}\left(X\right)\right)\right)\right)\right) \end{array}$$

where *X* is the input feature vector, *f*_*in*_ - a 2-layer MLP with {256, 256} features and GELU activation, *f*_*out*_ - linear layer with a single feature and no activation, *Pool*_*global*_ - global average pooling across nodes.

### 2.4 Training and inference procedures

#### 2.4.1 Loss function

We use the Pairwise Hinge Loss (PairwiseHingeLoss, Joachims, 2002; Agarwal et al., 2019) loss function for training the model.


(8) $$\begin{array}{l} L\left(\left\{r\right\},\left\{s\right\}\right)=\underset{i}{\sum }\underset{j}{\sum }I\left[r_{i}>r_{j}\right]\max\left(0,1-\left(s_{i}-s_{j}\right)\right) \end{array}$$

where *r*_*i*_ - are the ground truth runtimes, *s*_*i*_ - are the scores predicted by the model.

It is important that the predicted scores *s*_*i*_ = *R*_*neural*_(*c*_*i*_) do not correspond to the absolute values of runtimes *r*_*i*_ = *R*(*c*_*i*_). The applied loss function is a ranking loss function. It trains the model to order (rank) the predicted values in the same way as they are ordered by *R*(*c*). The correct ordering is enough to satisfy Equation 1.

#### 2.4.2 Training details

We train separate model instances for all collections. We've identified that separate models perform better than a joint model trained on all collections or models that were trained on all-xla or all-nlp combinations as well as all-random or all-default.

We use Adam (Kingma and Ba, 2014) optimizer (specifically AdamW version) with the learning rate of 1e-3, 0.05 of the total number of epochs as linear warm-up, a single-cycle (lifted cosine) learning rate schedule, and weight decay of 1e-5 for non-bias parameters. We apply gradient norm clipping at value 1.0.

We train the tile-xla collection for 17.5 epochs, whereas layout-nlp collections for 1000 epochs and layout-xla collections for 750 epochs.

Training wall-clock time is 2.5 hours per fold per collection measured on RTX4090 with 24 GB RAM. Training one set of models for all collections produces 13.45 kg CO_2_ as per Lacoste et al. (2019).

#### 2.4.3 Data splits

Whereas the official training/validation split is reasonably designed, we, however, employ K-fold cross-validation with *K* = 20 on the merged train/validation data splits. We train the first 5 folds to limit the training compute. We then pick the top-4 folds by the validation score to combat the instability of training. This choice comes from the slight instability of training: in rare cases, the training process for a specific fold may get stuck at a local minimum or experience partial parameter corruption due to gradient explosion. In addition, we choose not to split configurations of the same graph into train/validation since it would introduce a train-to-validation leak due to the very high correlation of configuration runtimes within the same graph.

### 2.5 Benchmark results

#### 2.5.1 Evaluation splits

TpuGraphs (Phothilimthana et al., 2023) dataset does not provide public test data annotations. Hence, we report the cross-validation score according to the Section 2.4.3.

#### 2.5.2 Evaluation metrics

Kendall's τ (Kendall's rank correlation coefficient) is used as the metric for layout collections:


(9) $$\begin{array}{l} \tau =\frac{2}{n\left(n-1\right)}\underset{i<j}{\sum }\text{sgn}\left(s_{i}-s_{j}\right)\text{sgn}\left(r_{i}-r_{j}\right) \end{array}$$

where *s* are the predicted scores, *r* are the ground truth runtimes, *n* is the batch size.

For the tile collection, the metric is set as:


(10) $$\begin{array}{l} M_{\text{tile}}=1-\left(\frac{\text{Best runtime of top-}k\;\text{predictions}}{\text{Best runtime of all configurations}}-1\right)\\ \;=2-\frac{\min_{i\in K}r_{i}}{\min_{i\in A}r_{i}} \end{array}$$

where *K* = 5.

#### 2.5.3 Details of the inference mode

For inference, we use the batch size of 128. However, since the prediction depends on the batch, we leverage the batch further by applying test-time augmentation (TTA) to generate N (10) permutations of the configurations and average the result after sorting it back to the original order. We average the scores of models trained on different folds.

The single-batch wall clock time is 60 ms on average for 1 fold and 240 ms on average for all 4 folds per collection.

#### 2.5.4 Experimental results

Our experimental results are summarized in the Table 2. The confidence ranges are reported as 1-sigma. We demonstrate state-of-the-art performance in 4 out of 5 collections. On xla-default Xu et al. (2023) show better results than our work; however, their results may contain an error since xla-default collection is harder than xla-random due to closer and harder-to-distinguish runtime annotations (the pattern is also followed by the results of TpuGraphs; Phothilimthana et al., 2023), but the score of Xu et al. (2023) for xla-default is higher than for xla-random which is very implausible.

**Table 2:** Experimental results.

**Collection**	**Metric**	**Validation score**		
		**TpuGraphs (Phothilimthana et al., 2023)**	**(Xu et al., 2023)**	**TGraph (ours)**
layout:xla:random	Kendall's τ	0.19	0.5285	**0.6840** ± 0.0110
layout:xla:default	Kendall's τ	0.12	**0.5887**	0.4785 ± 0.0031
layout:nlp:random	Kendall's τ	0.58	0.8387	**0.9713** ± 0.0008
layout:nlp:default	Kendall's τ	0.30	0.4841	**0.5628** ± 0.0027
mean across layout	Kendall's τ	0.298	0.610	**0.674**
tile:xla	*M*_*tile*_	–	0.8622	**0.9694** ± 0.0021

Row-wise best is highlighted with bold.

#### 2.5.5 Ablation study

Ablations for channel-wise self-attention, cross-configuration attention, and edges in the graph are collected in Table 3. While the effect of channel-wise self-attention is less obvious but nevertheless noticeable, the effect of cross-configuration attention is substantial, implying that the task of comparing the configurations between each other is easier than predicting the absolute values of runtimes. Additionally, we ablate the edges of the GraphSage GNN to demonstrate how essential the connectivity between the computational nodes is. In tensor compilers the adjacent operators are often fused into a single optimized operator, the procedure commonly know as kernel fusion. For a model solving the problem of predicting computational graph runtimes it is paramount to implicitly learn the “rules” of kernel fusion from data since the early stages of tensor compilation including kernel fusion are treated as a black box.

“*Data centers will use 8% of US power by 2030, compared with 3% in 2022.”*

– Sachs (2024)

**Table 3:** Ablation study.

**Configuration**	**Validation score, Kendall's** τ			
	**layout:xla:random**	**layout:xla:default**	**layout:nlp:random**	**layout:nlp:default**
Final, all features	**0.6840**	0.4785	**0.9713**	**0.5628**
- Channel-wise self-attention	0.6737 (−0.0103)	**0.4787** (+0.0002)	0.9680 (−0.0033)	0.5555 (−0.0073)
- Cross-configuration attention	0.6539 (−0.0301)	0.4518 (−0.0267)	0.9387 (−0.0326)	0.5436 (−0.0192)
- Graph edges	0.5022 (−0.1818)	0.3631 (−0.1154)	0.7751 (−0.1962)	0.3349 (−0.2279)

Column-wise best is highlighted with bold. Red indicates a decrease in a metric, green indicates an increase relative to our final result (“Final, all features”).

### 2.6 Environmental impact case study

According to Loten (2023) the total data center AI workload consumption in Northern Virginia (NV), VA, the US was 2132 MW in 2023. Thus, the annual data center energy consumption can be estimated as 18.6 million MWh. Considering the carbon footprint of energy production in NV of 0.3 tonne CO_2_ per MWh as per (Statista, 2022) the total annual CO_2_ emissions of NV data centers can be assessed as 5.58 mln tonnes CO_2_. From the authors of XTAT (Phothilimthana et al., 2021) we take 5% as a reference number for the runtime speed-up across a diverse dataset of 150 neural architectures. Speeding up AI workloads by 5% with the more efficient execution would reduce CO_2_ emissions by 275'000 tonnes CO_2_ yearly in NV alone. This is equivalent to the annual emissions of 36'000 households (approximately 50% of all NV households). Even though it is yet to be determined how to estimate the real acceleration of computation based on the values of Kendall's τ, we expect the effect to be similar or superior to XTAT (Phothilimthana et al., 2021).

## 3 Conclusion

The proposed novel TGraph neural network architecture establishes a state-of-the-art on the TpuGraphs dataset. A significant contribution to the performance comes from channel-wise self-attention and cross-configuration attention operations. The latter acts as one of the batch normalization techniques, allowing the exchange of information between individual samples, which improves performance in ranking problems.

In general, more efficient ML-based tensor compilation methods have a very positive societal impact. Firstly, they decrease energy consumption and CO_2_ emissions of data centers, consequently helping to fight climate change. Secondly, they help to free software engineers from the tedious labor of re-implementing lots of highly specialized computational kernels for the constant flow of hardware releases. Even though it may seem that it is a case of “AI taking over people's jobs”, in fact, the achieved extreme efficiency of digital infrastructure like data centers may cover the needs of people to the extent that they do not need to work or can opt to dedicate themselves to more human-centered activities.

## 4 Limitations and future work

The proposed neural network architecture is limited to predicting the runtimes of a static tensor program that can be represented as a computational graph. Another limitation is that the proposed method is not able to learn the behavior of the tensor program if the behavior is dependent on the values of input or intermediate data. As a machine learning algorithm, the proposed method requires a substantial amount of training data. In the absence of a diverse sample of benchmarked architectures, the domain gap between the training graphs and the unknown test graphs may be big enough, and the model is not able to generalize to it. The proposed method does not provide any guidance on how to choose the graphs for the creation of the training dataset. The proposed method does not generalize to unknown operators. New graphs with the new operator must be added to the training data in order for the model to learn the information about its contribution to the runtime. An ML model trained on one hardware (TPU) does not necessarily generalize to other hardware (GPU, CPU, etc) and must be re-trained for other hardware. Lastly, the proposed solution addresses two compilation sub-problems: tensor layout selection and tensor tiling selection, whereas there are more sub-problems to be solved by tensor compilers.

A potential future direction of research could be to transition from a predictive model to a generative model that is capable of directly proposing efficient configurations. This could involve training a variational autoencoder or diffusion model on the configuration space to generate novel tensor layouts and tiling strategies that may outperform configurations found through traditional search methods.

## Data Availability

Publicly available datasets were analyzed in this study. This data can be found here: https://www.kaggle.com/competitions/predict-ai-model-runtime/data.
